# Deficiency in DNA damage response of enterocytes accelerates intestinal stem cell aging in *Drosophila*

**DOI:** 10.18632/aging.101390

**Published:** 2018-03-07

**Authors:** Joung-Sun Park, Ho-Jun Jeon, Jung-Hoon Pyo, Young-Shin Kim, Mi-Ae Yoo

**Affiliations:** 1 Department of Molecular Biology, Pusan National University, Busan 46241, Republic of Korea; * Equal contribution

**Keywords:** *Drosophila*, enterocyte, niche, DNA damage response, intestinal stem cell aging

## Abstract

Stem cell dysfunction is closely linked to tissue and organismal aging and age-related diseases, and heavily influenced by the niche cells’ environment. The DNA damage response (DDR) is a key pathway for tissue degeneration and organismal aging; however, the precise protective role of DDR in stem cell/niche aging is unclear. The *Drosophila* midgut is an excellent model to study the biology of stem cell/niche aging because of its easy genetic manipulation and its short lifespan. Here, we showed that deficiency of DDR in *Drosophila* enterocytes (ECs) accelerates intestinal stem cell (ISC) aging. We generated flies with knockdown of *Mre11*, *Rad50*, *Nbs1*, *ATM*, *ATR*, *Chk1*, and *Chk2*, which decrease the DDR system in ECs. EC-specific DDR depletion induced EC death, accelerated the aging of ISCs, as evidenced by ISC hyperproliferation, DNA damage accumulation, and increased centrosome amplification, and affected the adult fly’s survival. Our data indicated a distinct effect of DDR depletion in stem or niche cells on tissue-resident stem cell proliferation. Our findings provide evidence of the essential role of DDR in protecting EC against ISC aging, thus providing a better understanding of the molecular mechanisms of stem cell/niche aging.

## Introduction

Stem cells play critical roles in the maintenance of tissue homeostasis, and their declining function is closely linked to tissue and organismal aging and age-related diseases [1,2]. Stem cells residing in niche microenvironments are surrounded by heterogeneous cell populations, and the importance of niches for stem cell functional integrity is well documented [1,2]. Therefore, exploration of the mechanisms of niches that accelerate the aging of tissue-resident stem cells would provide mechanistic insights into the regulation of tissue homeostasis, organismal aging, and age-related diseases, such as cancer.

The *Drosophila* midgut is a well-accepted model for aging studies, including stem cells/niches and aging-related changes because of its easy genetic manipulation and short lifespan [3–6]. *Drosophila* intestinal stem cells (ISCs) are the only mitotic cells in the adult midgut [3–5]. *Drosophila* ISCs generate two types of differentiated progeny: Absorptive polyploid enterocytes (ECs) and secretory enteroendocrine cells (EEs) via enteroblasts (EBs) [5]. These cell types are distinguished by the expression of cell-specific markers [3–5,7].

The intrinsic and extrinsic oxidative stresses caused by aging, infection, and high metabolism can activate ISC proliferation [8–13]. In aged and oxidative stressed guts, increased proliferation of ISC is linked to the accumulation of DNA damage and increased centrosome amplification, which are hallmarks of cancer [8,10,14–16]. In the regulation of ISC proliferation, internal pathways of ISCs such as Notch, Dome/JAK/STAT, EGFR, Pvf2/PVR, Hippo, InR, TOR, and Dpp/Tkv and paracrine factors such as Upds, Yki, Wg, Vn, Dilp3, and Dpp from ISC niches including ECs, EBs, EEs, and visceral muscles are involved [5,8,17–32].

ECs are constantly exposed to external factors and many extrinsic stresses and anti-cancer chemotherapies can induce the death of intestinal epithelial cells [33–35]. Recent studies have demonstrated that EC death is a major cause of accelerated ISC proliferation [9,12,33,36,37]. EC death is correlated with JNK activation during excessive stresses, such as infection or damaged conditions [9,38]. More recently, the mechanisms of tumor-host normal cell competition driving stem cell-derived tumor growth were delineated in this midgut model [39]. These findings clearly indicated the need for protective mechanisms of ECs that are essential for the maintenance of ISC quiescence (i.e., low dividing rate); however, such regulatory mechanisms of ECs for optimal ISC homeostatic maintenance are poorly understood at present.

For cell survival or death under diverse stresses, the DNA damage response (DDR) system is conserved from yeast to mammals [40,41]. DDR involves sensors including the MRE11/RAD50/NBS1 (MRN) complex, mediators including A-T mutated (ATM), A-T- and RAD3-related (ATR), DNA-dependent protein kinase (DNA-PK), and effectors including checkpoint 1 (CHK1) and CHK2 [40]. Expression of DDR-related factors is associated with the modulation of aging and diseases including cancer [42,43]. Interestingly, the extent of DDR’s influence on the regulation of the stem cell number and their proliferation are currently unknown, however, DDR suppression leads to stem cell loss in insects and mammals [44,45]. In humans, patients heterozygous for DDR-related genes affecting DDR system have an increased risk of cancer development. Cancer, an aging-related disease, is closely linked to the hyperproliferation activity of stem cells in stem cell-derived tumorigenesis [46–48]. In addition, patients with ataxia-telangiectasia generally die by the second or third decade of life [49]. We suspected that the different phenotypes in stem cell proliferation induced by DDR deficiency might be associated with differences in the depletion in stem and niche cells. Although the depletion of stem cell-intrinsic DDR leads to decreased proliferation activity and loss of stem cells [45] and DDR increases in ECs undergoing age and oxidative stress [14,45], the role of niche-specific DDR in aging of tissues-resident stem cells has not been clearly demonstrated.

In the present study, using flies with EC-specific knockdown of DDR-related genes, we attempt to determine the protective role of DDR in differentiated ECs during ISC aging.

## RESULTS

### Requirement of DDR-related factors in DDR of EC

To investigate the requirement of DDR-related factors in DDR of EC, we generated flies with EC-specific knockdown of *Mre11*, *Rad50*, *Nbs1*, *ATM*, *ATR*, *Chk1*, and *Chk2*, which are sensors, mediators, or effectors of the DDR system, using flies with the *Myo^ts^>GFP* genotype. DDR directs a cell to repair DNA double-strand breaks (DSBs), a major driver of intrinsic aging. γH2AX is a dependable indicator of DNA damage response [50,51]. To determine the activation of DDR in ECs when exposed to DNA damage, we examined the signal strength of γH2AvD, analogous to mammal γH2AX, in Myo-GFP^+^ cells of the gut from *Myo^ts^>GFP* flies 1 h after the application of 5 Gy of γ-ray irradiation as an inducer of the DNA damage. While week γH2AvD signals were detected in ECs and Myo-GFP^-^ cells (ISCs, EBs, and EEs) in the non-irradiated *Myo^ts^>GFP* wild-type flies (Fig. 1A a-a’, yellow arrow), strong γH2AvD signals were detected in ECs and in Myo-GFP^-^ cells (ISCs, EBs, and EEs) in the irradiated *Myo^ts^>GFP* wild-type flies (Fig. 1A i-i’, yellow arrow). This indicates the activation of DDR in EC against DNA damage. To determine the requirement of DDR-related factors in DNA damage-induced DDR activation in ECs, we examined the signal strength of γH2AvD in Myo-GFP^+^ cells of the gut from *Myo^ts^>GFP+Mre11i*, *Myo^ts^>GFP+Rad50i*, *Myo^ts^>GFP+Nbs1i*, *Myo^ts^>GFP+ATMi*, *Myo^ts^>GFP+ATRi*, *Myo^ts^>GFP+Chk1i*, and *Myo^ts^>GFP+Chk2i* flies 1 h after irradiation. In contrast to the signal in wild-type *Myo^ts^>GFP* flies, the γ-irradiation-induced increase in the γH2AvD signal was greatly reduced in Myo-GFP^+^ cells (ECs) of *Myo^ts^>GFP+Mre11i*, *Myo^ts^>GFP+Rad50i*, *Myo^ts^>GFP+Nbs1i*, *Myo^ts^>GFP+ATMi*, *Myo^ts^>GFP+ATRi*, *Myo^ts^>GFP+Chk1i*, and *Myo^ts^>GFP+Chk2i* flies (Fig. 1A j-p’, yellow arrow). At this time point, strong γH2AvD signals were detected in Myo-GFP^-^ cells (ISCs, EBs, and EEs) from the irradiated *Myo^ts^>GFP*, *Myo^ts^>GFP+Mre11i*, *Myo^ts^>GFP+Rad50i*, *Myo^ts^>GFP+Nbs1i*, *Myo^ts^>GFP+ATMi*, *Myo^ts^>GFP+ATRi*, *Myo^ts^>GFP+Chk1i*, and *Myo^ts^>GFP+Chk2i* flies (Fig. 1A j-p’). These results indicated that the EC-specific knockdown of DDR-related factors specifically affected the activation of DDR system in ECs.

**Figure 1A:**
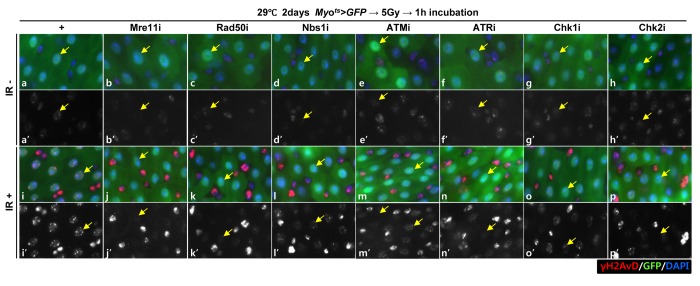
**EC-specific knockdown of DDR cause EC cell death.** Effect of knockdown of EC-specific DDR-related factors on γH2AvD signals after irradiation. γH2AvD signals after 5 Gy irradiation in the EC-specific Mre11, Rad50, Nbs1, ATM, ATR, Chk1, or Chk2 knockdown in the midgut. Flies carrying *Myo^ts^>GFP* (**a**-**a’** and i-I’), *Myo^ts^>GFP*+*Mre11i* (**b**-**b’** and **j**-**j’**), *Myo^ts^>GFP+Rad50i* (**c**-**c’** and **k**-**k’**), *Myo^ts^>GFP*+*Nbs1i* (**d**-**d’** and **l**-**l’**), *Myo^ts^>GFP*+*ATMi* (**e**-**e’** and **m**-**m’**), *Myo^ts^>GFP*+*ATRi* (**f**-**f’** and **n**-**n’**), *Myo^ts^>GFP*+*Chk1i* (**g**-**g’** and **o**-**o’**), or *Myo^ts^>GFP*+*Chk2i* (**h**-**h’** and **p**-**p’**) were cultured at 29 °C for 2 days and exposed to γ-radiation. **a**-**h’**, non-irradiation. **i**-**p’**, 5 Gy irradiation. **a**-**p** panels depict the merged images and **a’**-**p’** panels denote the gray scale versions for the images corresponding to γH2AvD signals. One hour after irradiation, the guts of the irradiated flies were dissected and labeled with anti-GFP (green) and anti-γH2AvD (red) antibodies and 4′,6-diamidino-2-phenylindole (DAPI, blue). Yellow arrows indicate EC. Original magnification is 400×.

### Knockdown of DDR-related factors in the EC induces EC death

To assess the role of DDR-related factors in EC death, we examined Cleaved caspase-3 signals and found that they were increased in the Myo-GFP^+^ cells of the gut from *Myo^ts^>GFP*, *Myo^ts^>GFP+Mre11i*, *Myo^ts^>GFP+Rad50i*, *Myo^ts^>GFP+Nbs1i*, *Myo^ts^>GFP+ATMi*, *Myo^ts^>GFP+ATRi*, *Myo^ts^>GFP+Chk1i*, and *Myo^ts^>GFP+Chk2i* flies kept at 29 °C for 4 days. Very weak Cleaved caspase-3 signals were detected in ECs in *Myo^ts^>GFP* wild-type flies (Fig. 1B a-a’). In contrast to the signal in the wild-type *Myo^ts^>GFP* flies, the Cleaved caspase-3 signal was greatly increased in Myo-GFP^+^ cells (ECs) of *Myo^ts^>GFP+Mre11i*, *Myo^ts^>GFP+Rad50i*, *Myo^ts^>GFP+Nbs1i*, *Myo^ts^>GFP+ATMi*, *Myo^ts^>GFP+ATRi*, *Myo^ts^>GFP+Chk1i*, and *Myo^ts^>GFP+Chk2i* flies (Fig. 1B b-h’). We quantified the ratio of Cleaved caspase-3^+^ in Myo-GFP^+^ cells. Significant increases of EC death were detected in the gut of EC-specific DDR-related factor knockdown (Fig. 1B i). In the Myo-GFP^-^ small cells of the gut from *Myo^ts^>GFP*, *Myo^ts^>GFP+Mre11i*, *Myo^ts^>GFP+Rad50i*, *Myo^ts^>GFP+Nbs1i*, *Myo^ts^>GFP+ATMi*, *Myo^ts^>GFP+ATRi*, *Myo^ts^>GFP+Chk1i*, and *Myo^ts^>GFP+Chk2i*, signals of Cleaved caspase-3 were not detected (Fig. 1B). EC-specific DDR knockdown-induced ECs death could be suppressed by coexpression of the Caspase inhibitor, DIAP1 (Suppl. Fig. 1 and 2), indicating that DDR knockdown induced ECs death.

**Figure 1B:**
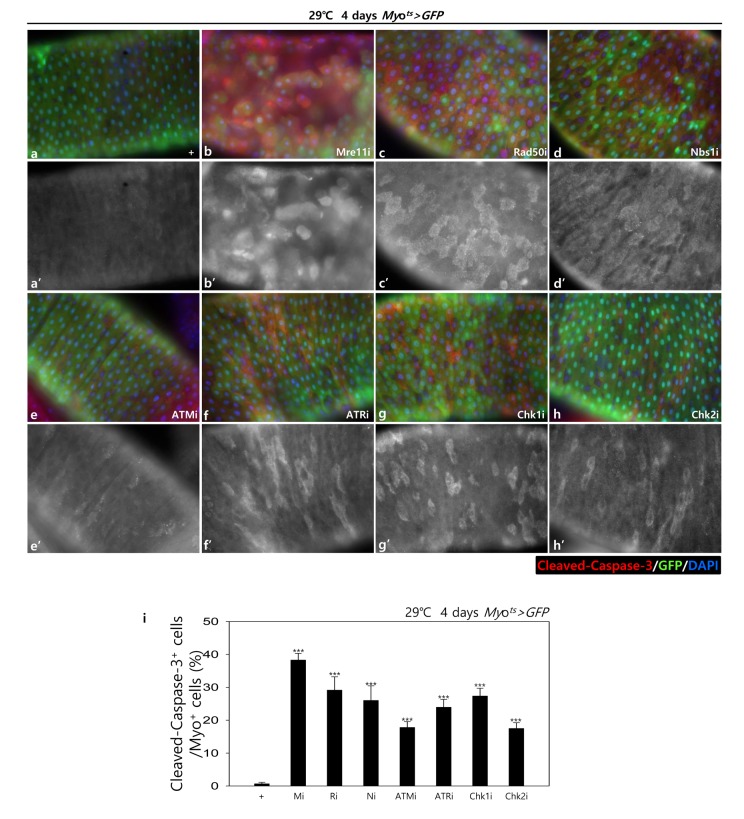
**EC-specific knockdown of DDR cause EC cell death.** EC-specific knockdown of Mre11, Rad50, Nbs1, ATM, ATR, Chk1, or Chk2 induce Cleaved caspase-3 in EC. Flies carrying *Myo^ts^>GFP* (**a**-**a’**), *Myo^ts^>GFP*+*Mre11i* (**b**-**b’**), *Myo^ts^>GFP+Rad50i* (**c**-**c’**), *Myo^ts^>GFP*+*Nbs1i* (**d**-**d’**), *Myo^ts^>GFP*+*ATMi* (**e**-**e’**), *Myo^ts^>GFP*+*ATRi* (**f**-**f’**), *Myo^ts^>GFP*+*Chk1i* (**g**-**g’**), or *Myo^ts^>GFP*+*Chk2i* (**h**-**h’**) genotypes were cultured at 29 °C for 4 days. **a**-**h** panels depict the merged images and **a’**-**h’** panels denote the gray scale versions for the images corresponding to Cleaved caspase-3 signals. Original magnification is 400×. (**i**) A graph showing the ratio of Cleaved caspase-3^+^ cells in Myo-GFP^+^ cells. The data (mean ± SE) from 5 guts, respectively. ***p < 0.0001. The guts of flies were dissected and labeled with anti-GFP (green) and anti-Cleaved caspase-3 (red) antibodies and DAPI (blue).

In addition, to check the role of DDR-related factors on the activation of the JNK signal in EC cells, pJNK signals were examined in Myo-GFP^+^ cells of the gut from *Myo^ts^>GFP*, *Myo^ts^>GFP+Mre11i*, *Myo^ts^>GFP+Rad50i*, *Myo^ts^>GFP+Nbs1i*, *Myo^ts^>GFP+ATMi*, *Myo^ts^>GFP+ATRi*, *Myo^ts^>GFP+Chk1i*, and *Myo^ts^>GFP+Chk2i* flies kept at 29 °C for 4 days. Very weak signals of pJNK, a cell death marker, were detected in ECs in *Myo^ts^>GFP* wild-type flies (Fig. 1C a-a’). By contrast, the pJNK signal was greatly increased in the Myo-GFP^+^ cells (ECs) of *Myo^ts^>GFP+Mre11i*, *Myo^ts^>GFP+Rad50i*, *Myo^ts^>GFP+Nbs1i*, *Myo^ts^>GFP+ATMi*, *Myo^ts^>GFP+ATRi*, *Myo^ts^>GFP+Chk1i*, and *Myo^ts^>GFP+Chk2i* flies (Fig. 1C b-h’). We quantified the ratio of pJNK^+^ in Myo-GFP^+^ cells. The significant increases of EC death were detected in the gut of EC-specific DDR-related factor knockdown (Fig. 1C i). These results indicated that DDR-related factors are required for EC survival in normal conditions.

**Figure 1C:**
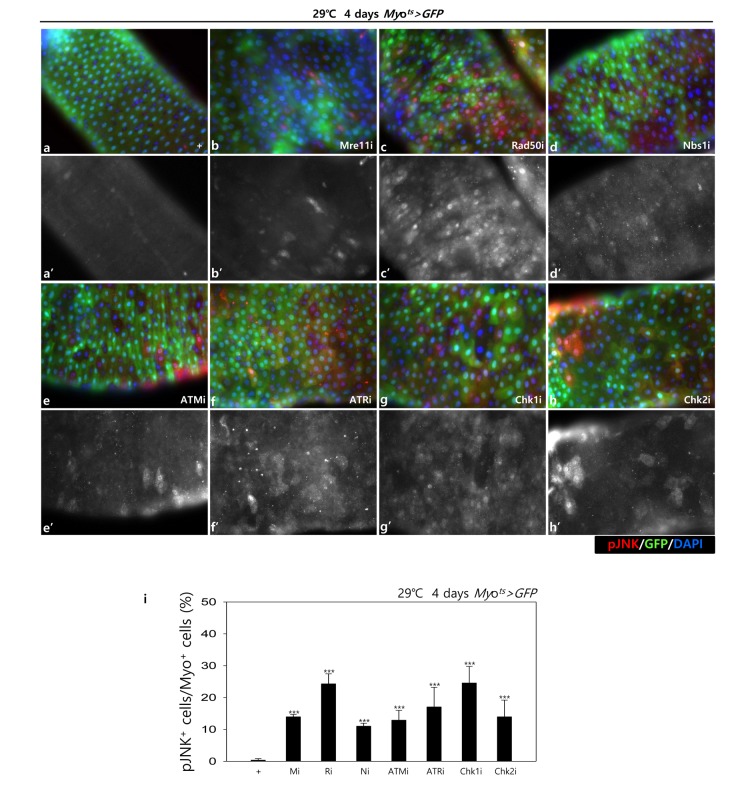
**EC-specific knockdown of DDR cause EC cell death.** EC-specific knockdown of Mre11, Rad50, Nbs1, ATM, ATR, Chk1, or Chk2 induce JNK activation in ECs. Flies carrying *Myo^ts^>GFP* (**a**-**a’**), *Myo^ts^>GFP*+*Mre11i* (**b**-**b’**), *Myo^ts^>GFP+Rad50i* (**c**-**c’**), *Myo^ts^>GFP*+*Nbs1i* (**d**-**d’**), *Myo^ts^>GFP*+*ATMi* (**e**-**e’**), *Myo^ts^>GFP*+*ATRi* (**f**-**f’**), *Myo^ts^>GFP*+*Chk1i* (**g**-**g’**), or *Myo^ts^>GFP*+*Chk2i* (**h**-**h’**) genotypes were cultured at 29°C for 4 days. **a**-**h** panels depict the merged images and **a’**-**h’** panels denote the gray scale versions for the images corresponding to pJNK signals. Original magnification is 400×. (**i**) A graph showing the ratio of pJNK^+^ cells in Myo-GFP^+^ cells. The data (mean ± SE) from 5 guts, respectively. ***p < 0.0001. The guts of flies were dissected and labeled with anti-GFP (green) and anti-pJNK (red) antibodies and DAPI (blue).

### Knockdown of DDR-related factors in EC induces ISC aging

**Figure 2:**
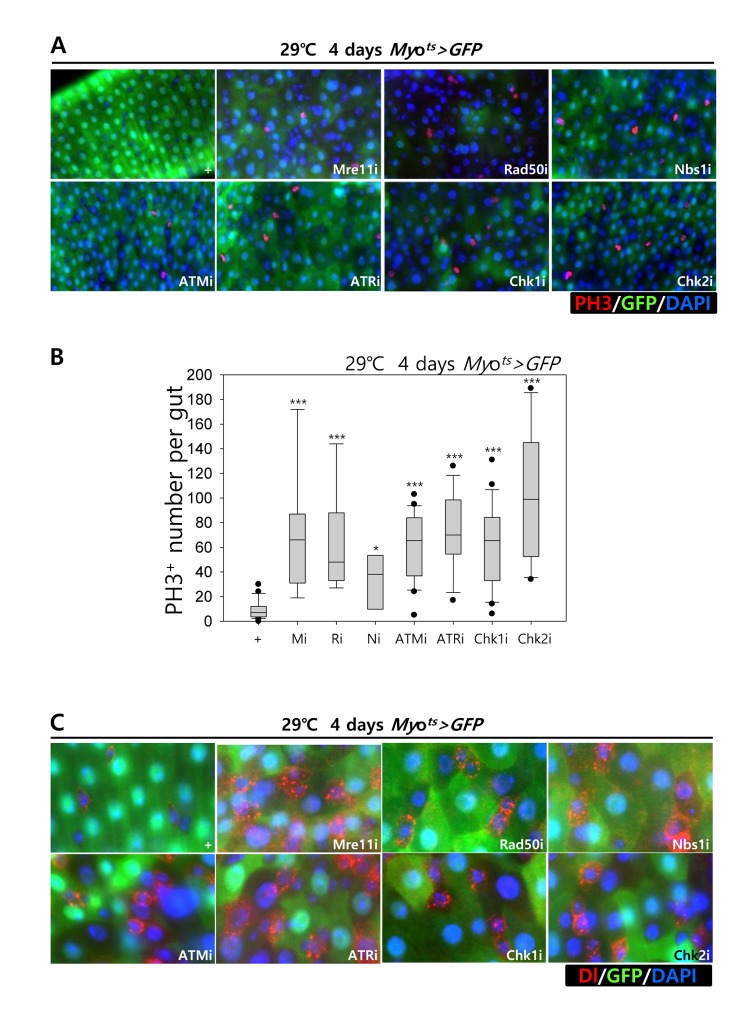
**Effects of the knockdown of EC-specific DNA damage response (DDR)-related factors on ISC proliferation.** (**A**-**B**) EC-specific knockdown of Mre11, Rad50, Nbs1, ATM, ATR, Chk1, or Chk2 induce ISC division. Flies carrying *Myo^ts^>GFP*, *Myo^ts^>GFP*+*Mre11i*, *Myo^ts^>GFP+Rad50i*, *Myo^ts^>GFP*+*Nbs1i*, *Myo^ts^>GFP*+*ATMi*, *Myo^ts^>GFP*+*ATRi*, *Myo^ts^>GFP*+*Chk1i*, or *Myo^ts^>GFP*+*Chk2i* genotypes were cultured at 29 °C for 4 days. The guts of flies were dissected and labeled with anti-GFP (green) and anti-PH3 (red) antibodies and DAPI (blue). Original magnification is 400×. (**B**) A graph showing the PH3^+^ cell number in the midgut with an EC-specific knockdown of Mre11, Rad50, Nbs1, ATM, ATR, Chk1, or Chk2. The gut specimens of *Myo^ts^>GFP*, *Myo^ts^>GFP*+*Mre11i*, *Myo^ts^>GFP+Rad50i*, *Myo^ts^>GFP*+*Nbs1i*, *Myo^ts^>GFP*+*ATMi*, *Myo^ts^>GFP*+*ATRi*, *Myo^ts^>GFP*+*Chk1i*, or *Myo^ts^>GFP*+*Chk2i* flies (kept at 29 °C for 4 days) were labeled with anti-GFP (green) and anti-PH3 (red) antibodies and DAPI (blue). The numbers of PH3^+^ cells were counted in the whole gut under a microscope. Data (mean±SE) in *Myo^ts^>GFP*, *Myo^ts^>GFP*+*Mre11i*, *Myo^ts^>GFP+Rad50i*, *Myo^ts^>GFP*+*Nbs1i*, *Myo^ts^>GFP*+*ATMi*, *Myo^ts^>GFP*+*ATRi*, *Myo^ts^>GFP*+*Chk1i*, or *Myo^ts^>GFP*+*Chk2i* flies were collated from 21, 22, 13, 20, 9, 9, 26, and 10 guts, respectively. *p*-values were calculated using student’s *t*-test. *p < 0.01, ***p < 0.0001. (**C**) EC-specific knockdown of Mre11, Rad50, Nbs1, ATM, ATR, Chk1, or Chk2 increased the number of Delta-positive cells. Flies carrying *Myo^ts^>GFP*, *Myo^ts^>GFP*+*Mre11i*, *Myo^ts^>GFP+Rad50i*, *Myo^ts^>GFP*+*Nbs1i*, *Myo^ts^>GFP*+*ATMi*, *Myo^ts^>GFP*+*ATRi*, *Myo^ts^>GFP*+*Chk1i*, or *Myo^ts^>GFP*+*Chk2i* genotypes were cultured at 29 °C for 4 days. The guts of flies were dissected and labeled with anti-GFP (green) and anti-Delta (red) antibodies and DAPI (blue). Original magnification is 400×.

Furthermore, we examined whether the knockdown of DDR-related factors in the EC affects ISC proliferation using anti-PH3 (a marker of mitotic cells) and anti-Delta (a marker of intestinal stem cell) antibodies. These gene knockdowns were assessed in ECs using *Myo^ts^>GFP* flies kept at 29 °C for 4 days. As expected, a dramatic increase in ISC proliferation was detected in the guts of *Myo^ts^>GFP+Mre11i*, *Myo^ts^>GFP+Rad50i*, *Myo^ts^>GFP+Nbs1i*, *Myo^ts^>GFP+ATMi*, *Myo^ts^>GFP+ATRi*, *Myo^ts^>GFP+Chk1i*, and *Myo^ts^>GFP+Chk2i* flies compared with that in the control (Fig. 2A). The number of PH3^+^ cells significantly increased in guts harboring the EC-specific knockdown of DDR-related factors (Fig. 2B). In addition, the number of Delta^+^ cells also dramatically increased in guts with EC-specific knockdown of DDR-related factors (Fig. 2C). These results indicated that the loss of DDR-related factors in ECs induced ISC hyperproliferation.

To assess the implication of excessive ISC proliferation by EC-specific DDR-related factor knockdown-induced EC death, we analyzed the DNA damage accumulation in ISCs using an anti-γH2AvD antibody, a molecular marker of the DSBs [14,52], and anti-Delta antibody. The γH2AvD signal was very low in the Myo-GFP^-^ and Delta^+^ cells (a marker of ISCs) of *Myo^ts^>GFP* flies (Fig. 3A a-a’); however, γH2AvD foci were dramatically increased in the Myo-GFP^-^ and Delta^+^ cells (ISCs) of *Myo^ts^>GFP+Mre11i*, *Myo^ts^>GFP+Rad50i*, *Myo^ts^>GFP+Nbs1i*, *Myo^ts^>GFP+ATMi*, *Myo^ts^>GFP+ATRi*, *Myo^ts^>GFP+Chk1i*, and *Myo^ts^>GFP+Chk2i* flies (Fig. 3A b-h’). These results indicated that the EC-specific knockdown of DDR-related factors could induce DNA damage accumulation in ISCs. Furthermore, EC-specific DDR knockdown-induced DNA damage accumulation in ISCs could be suppressed by coexpression of the DIAP1 (Suppl. Fig. 3), indicating that EC-specific DDR knockdown-induced ISC aging is associated with EC death. We also checked the centrosome amplification (a hallmark of cancer cells) using anti-γ-tubulin and anti-PH3 antibodies. In control files, two centrosomes in the mitotic ISCs (PH3^+^ cells) were detected; however, mitotic ISCs with 3–12 abnormal centrosomes were detected in the EC-specific DDR-related factor knockdown flies carrying *Myo^ts^>GFP+Mre11i*, *Myo^ts^>GFP+Rad50i*, *Myo^ts^>GFP+Nbs1i*, *Myo^ts^>GFP+ATMi*, *Myo^ts^>GFP+ATRi*, *Myo^ts^>GFP+Chk1i*, and *Myo^ts^>GFP+Chk2i* genotypes (Fig. 3B a). We quantified the frequencies of these mitotic ISCs with supernumerary centrosomes (>2), which were 9.4% in the *Myo^ts^>GFP+Mre11i* flies (N = 15, n = 449, N indicates the number of guts, n indicates the number of PH3^+^ cells), 12.2% in the *Myo^ts^>GFP+Rad50i* flies (N = 11, n = 557), 6.8% in the *Myo^ts^>GFP+Nbs1i* flies (N = 15, n = 412), 14.6% in the *Myo^ts^>GFP+ATMi* flies (N = 13, n = 560), 8.7% in the *Myo^ts^>GFP+ATRi* flies (N = 16, n = 447), 9.6% in the *Myo^ts^>GFP+Chk1i* flies (N = 15, n = 687), 13.2% in the *Myo^ts^>GFP+Chk2i* flies (N = 9, n = 349), and 1.6% in the *Myo^ts^>GFP* flies (N = 15, n = 61) (Fig. 3B c). The number of mitotic ISCs with supernumerary centrosomes (>2) per gut was 2.8 in the *Myo^ts^>GFP+Mre11i* flies, 6.2 in the *Myo^ts^>GFP+Rad50i* flies, 1.9 in the *Myo^ts^>GFP+Nbs1i* flies, 6.3 in the *Myo^ts^>GFP+ATMi* flies, 2.4 in the *Myo^ts^>GFP+ATRi* flies, 4.4 in the *Myo^ts^>GFP+Chk1i* flies, 5.1 in the *Myo^ts^>GFP+Chk2i* flies, and 0.07 in the *Myo^ts^>GFP* flies (Fig. 3B d). These results show that the inhibition of DDR resulted in DNA damage accumulation and in a higher incidence of centrosome amplification in ISCs.

**Figure 3A:**
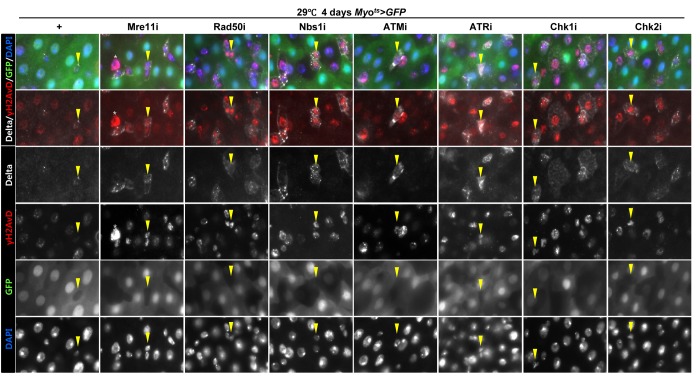
**EC-specific knockdown of DNA damage response (DDR)-related factors causes an increase in the age-related phenotypes of ISCs.** EC-specific knockdown of Mre11, Rad50, Nbs1, ATM, ATR, Chk1, or Chk2 induce DNA damage accumulation in ISCs. Flies carrying *Myo^ts^>GFP*, *Myo^ts^>GFP*+*Mre11i*, *Myo^ts^>GFP+Rad50i*, *Myo^ts^>GFP*+*Nbs1i*, *Myo^ts^>GFP*+*ATMi*, *Myo^ts^>GFP*+*ATRi*, *Myo^ts^>GFP*+*Chk1i*, or *Myo^ts^>GFP*+*Chk2i* genotypes were cultured at 29 °C for 4 days. The guts of flies were dissected and labeled with anti-GFP (green), anti-Delta (white), and anti-γH2AvD (red) antibodies and DAPI (blue). Yellow arrow heads indicate Delta^+^ cell. Upper two panels is merged image. Lower four panels is gray scale image of upper images. Asterisk indicates Myo^-^, Delta^-^, and strong γH2AvD^+^ cell, shows dying cell.

**Figure 3B:**
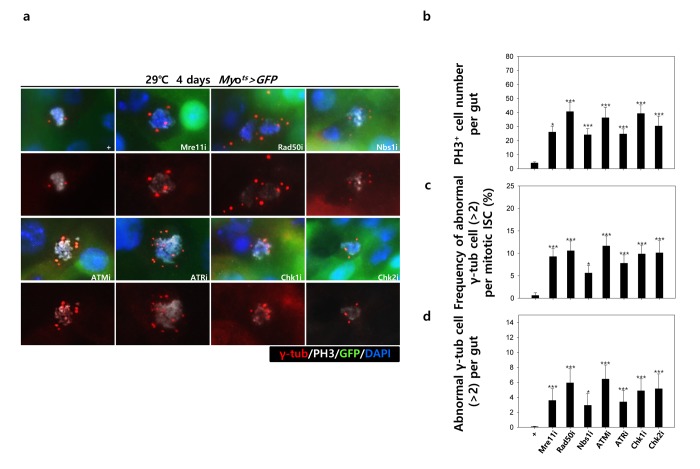
**EC-specific knockdown of DNA damage response (DDR)-related factors causes an increase in the age-related phenotypes of ISCs.** EC-specific knockdown of DDR-related factors cause centrosome amplification in ISCs. Flies carrying *Myo^ts^>GFP*, *Myo^ts^>GFP*+*Mre11i*, *Myo^ts^>GFP+Rad50i*, *Myo^ts^>GFP*+*Nbs1i*, *Myo^ts^>GFP*+*ATMi*, *Myo^ts^>GFP*+*ATRi*, *Myo^ts^>GFP*+*Chk1i*, or *Myo^ts^>GFP*+*Chk2i* genotypes were cultured at 29 °C for 4 days. (a) The guts of flies were dissected and labeled with anti-GFP (green), anti-γ-tubulin (red), and anti-PH3 (white) antibodies and DAPI (blue). Original magnification is 400×. (b-d) Increased number of mitotic ISCs with supernumerary centrosomes (>2) in the guts of *Myo^ts^>GFP*, *Myo^ts^>GFP*+*Mre11i*, *Myo^ts^>GFP+Rad50i*, *Myo^ts^>GFP*+*Nbs1i*, *Myo^ts^>GFP*+*ATMi*, *Myo^ts^>GFP*+*ATRi*, *Myo^ts^>GFP*+*Chk1i*, or *Myo^ts^>GFP*+*Chk2i* flies. (b) EC-specific knockdown of Mre11, Rad50, Nbs1, ATM, ATR, Chk1, or Chk2 cause the increase of mitotic ISCs in the midguts. (c) Frequency of abnormal γ-tubulin cell per mitotic ISC. (d) Number of abnormal γ-tubulin cell per midgut. Three-day-old females were shifted to 29 °C for 4 days and dissected guts were immunostained with anti-GFP (green), anti-γ-tubulin (red), and anti-PH3 (white) antibodies and DAPI (blue). The centrosome numbers were counted in the PH3^+^ cells of these guts. Data (mean±SE) in *Myo^ts^>GFP*, *Myo^ts^>GFP*+*Mre11i*, *Myo^ts^>GFP+Rad50i*, *Myo^ts^>GFP*+*Nbs1i*, *Myo^ts^>GFP*+*ATMi*, *Myo^ts^>GFP*+*ATRi*, *Myo^ts^>GFP*+*Chk1i*, or *Myo^ts^>GFP*+*Chk2i* flies were collated from 61, 449, 557, 412, 560, 447, 687, and 349 mitotic cells of 15, 15, 11, 15, 13, 16, 15, and 9 guts, respectively. *p*-values were calculated using student’s *t*-test. *p<0.001, ***p<0.0001 compared to that of the *Myo^ts^>GFP* flies.

Collectively, the results indicated that the knockdown of EC-specific DDR-related factors induced age-related phenotypes of ISCs, ISC hyperproliferation, DNA damage accumulation, and a higher incidence of centrosome amplification.

### Effect of knockdown of DDR-related factors in ECs at the organismal level

To further investigate the role of DDR-related factors at the organismal level, we checked whether EC-specific knockdown of DDR-related factors affected the adult fly’s survival. In the early stage of life, the survival of *Myo^ts^>GFP+Mre11i*, *Myo^ts^>GFP+Rad50i*, *Myo^ts^>GFP+Nbs1i*, *Myo^ts^>GFP+ATMi*, *Myo^ts^>GFP+ATRi*, *Myo^ts^>GFP+Chk1i*, and *Myo^ts^>GFP+Chk2i* females were significantly reduced compared to that of *Myo^ts^>GFP* females (Fig. 4A). Moreover, we assessed whether EC-specific DDR knockdown affected the response of ISCs to mild stress using an anti-PH3 antibody (a marker of dividing cells). Under 2 mM paraquat (PQ) fed conditions, ISC proliferation was highly increased in the guts of *Myo^ts^>GFP+Mre11i*, *Myo^ts^>GFP+Rad50i*, *Myo^ts^>GFP+Nbs1i*, *Myo^ts^>GFP+ATMi*, *Myo^ts^>GFP+ATRi*, *Myo^ts^>GFP+Chk1i*, and *Myo^ts^>GFP+Chk2i* flies, while it did not change in the guts of *Myo^ts^>GFP* flies (Fig. 4B). These results indicated that the guts with EC-specific knockdown of DDR-related factors are more sensitive to mild stress compared with that of wild-type flies.

**Figure 4A:**
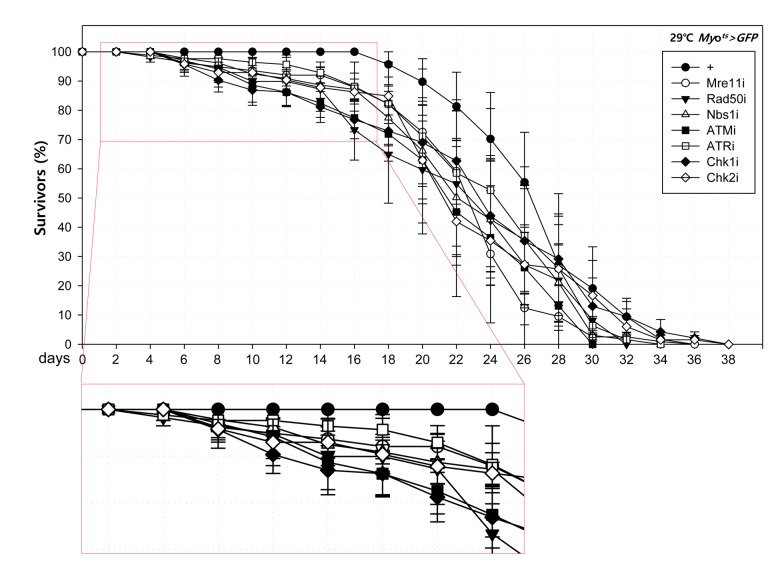
**High sensitivity to mild oxidative stresses exhibited by flies with EC-specific knockdown of DNA damage response (DDR)-related factors.** Death rate at the early stage of flies with the knockdown of EC-specific DDR-related factors. Flies carrying *Myo^ts^>GFP* (closed circle), *Myo^ts^>GFP*+*Mre11i* (open circle), *Myo^ts^>GFP+Rad50i* (closed inverted triangle), *Myo^ts^>GFP*+*Nbs1i* (open triangle), *Myo^ts^>GFP*+*ATMi* (closed quadrangle), *Myo^ts^>GFP*+*ATRi* (open quadrangle), *Myo^ts^>GFP*+*Chk1i* (closed rhombus), or *Myo^ts^>GFP*+*Chk2i* (open rhombus) genotypes were cultured at 29 °C and survivors were counted every two days (n=62, 82, 56, 58, 77, 92, 78, 59, respectively).

**Figure 4B:**
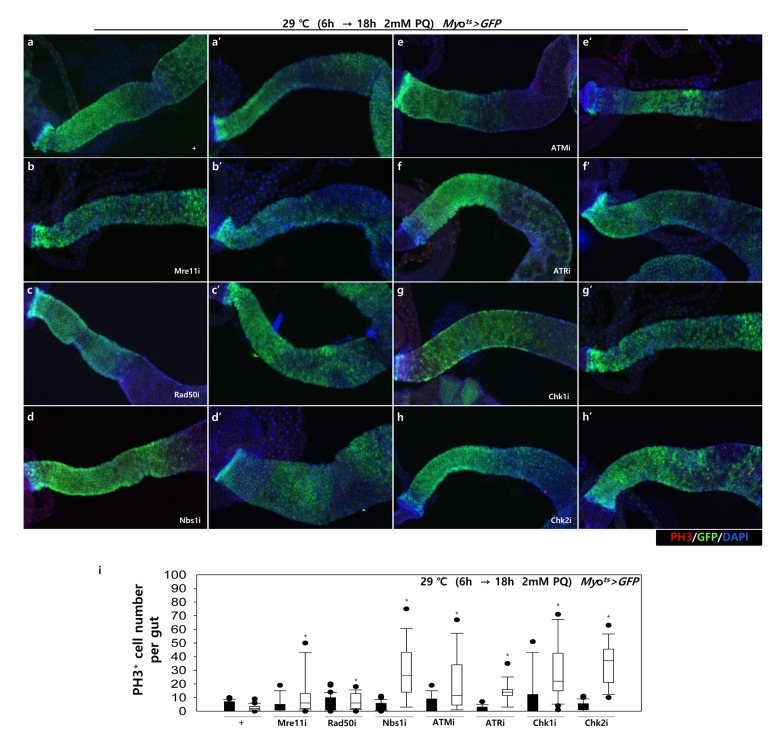
**High sensitivity to mild oxidative stresses exhibited by flies with EC-specific knockdown of DNA damage response (DDR)-related factors.** EC-specific DDR-related factor knockdown flies evince a higher sensitivity to mild oxidative stress. Three-day-old flies carrying *Myo^ts^>GFP*, *Myo^ts^>GFP*+*Mre11i*, *Myo^ts^>GFP+Rad50i*, *Myo^ts^>GFP*+*Nbs1i*, *Myo^ts^>GFP*+*ATMi*, *Myo^ts^>GFP*+*ATRi*, *Myo^ts^>GFP*+*Chk1i*, or *Myo^ts^>GFP*+*Chk2i* genotypes were cultured for 6 h at 29 °C in normal media, and then were fed without (**a**-**h**, closed bars) or with 2 mM PQ (**a’**-**h’**, open bars) in 5% sucrose for 18 h at 29 °C. The number of PH3^+^ cells in their guts was imaged (**a**-**h’**) and counted (**i**). *p*-values were calculated using Student’s *t*-test. *p < 0.05.

## DISCUSSION

The present study demonstrated, for the first time, that depletion of EC-specific factors involved in DDR accelerated the ISC aging process, as shown by ISC hyperproliferation, DNA damage accumulation, and increased centrosome amplification, and affected the adult fly’s survival.

Our data indicated that the EC-specific DDR-knockdown activates apoptotic signals such as Cleaved caspase-3 and pJNK in ECs, demonstrating that DDR is required for the survival of ECs under normal conditions. Several possibilities exist for the need of DDR in EC survival: 1) ECs are constantly exposed to oxidative stress from external factors including food and microbiota [53]. Our data showed that when exposed to oxidative stress via a low dose of PQ as a mild stress condition, the guts with EC-specific knockdown of DDR-related factors were more sensitive to the induction of ISC proliferation than those of wild-type cells. 2) DDR may play an essential role in protecting against DNA replication stress in ECs because of endoreplication during differentiation. Several studies reported that DNA replication stress is one of the major factors causing DNA strand breaks [54]. The *Drosophila* midgut undergoes endoreplication during EB-to-EC differentiation [55–57]. In this study, we knocked down the DDR-related factors in mature ECs using *Myo^ts^-GAL4*; therefore, we aimed to examine whether DDR is involved in the repair of endoreplication-induced DNA replication stress during EC maturation. 3) ECs might need DDR to repair DNA damages generated from the genomic instability of ISCs. The requirement for the DDR system in ECs may increase with age, because the accumulation of age-related DNA damage was reported in *Drosophila* ISCs and in the intestinal crypt of aged mice [14,15,58].

In the present study, we showed that the knockdown of DDR-related factors in mature ECs induced ISC hyperproliferation. We previously reported that the ISC/EB-specific knockdown of Atm and Atr leads to loss of ISCs [45]. These data indicate a distinct effect of DDR deficiency in ISCs/EBs and differentiated ECs on ISC proliferation. Many patients with DNA repair disorders are characterized by progressive cerebellar degeneration, telangiectasia, immunodeficiency, and premature aging [59]. These phenotypes may be associated with reduced proliferation of tissue-resident stem cells [59,60]. By contrast, a recent study also reported that *Atm* mutation carriers have an increased risk of developing cancer, such as cancer of the breast and digestive tract [46]. Our data suggest that these different phenotypes of patients with DNA repair disorders might be associated with the distinct effect of DDR loss in stem cells and differentiated niche cells on stem cell proliferation.

Recently, several studies reported EC death in flies as the major cause for accelerating ISC proliferation [35,36,38]. Our data confirmed the findings that EC death accelerates ISC proliferation. Our data further showed that the knockdown of DDR-related factors in mature ECs induces centrosome amplification in mitotic ISCs. Previously, we reported centrosome amplification as a marker of aging ISCs [16], which may be induced by DNA damage [61]. In the present study, we showed that the knockdown of DDR-related factors in ECs induces DNA damage accumulation in ISCs.

Mammalian intestinal stem cells renew continually throughout life; therefore, their DDR activity in intestinal enterocytes could be more important for ISC homeostasis compared with that of insects. It is noteworthy that ISC proliferation in the intestine is linked to the lifespan of the organism [62]. Early age decline and shortened lifespan is reportedly in flies with mutations in *tefu* (ortholog of mammalian ATM) or *mei-41* (orthologue of mammalian ATR) compared to that in the wild-type [63,64]. The present study showed that the knockdown of DDR-related factors in mature ECs induced ISC hyperproliferation and affected the flies’ survival. This new finding is interesting in view of our previous data showing the ISC/EB-specific knockdown of ATM/ATR decreased ISC proliferation and reduced the flies’ survival [44].

In the present study, we found EC-specific knockdown of DDR-related factors affected differently the level of γH2AvD, cell death, and ISC aging phenotype. It was reported that ATR is more important than ATM on DDR in the ISCs [45]. In proliferating cells as a larval brain, *grp* (Chk1) mutant does not show severe defects in the DNA repair unlike *mei-41* (ART) mutant [65]. Further study needs to be done on which DDR-related factors play a more critical role in EC.

In summary, this study demonstrated that the inhibition of the DDR in differentiated ECs induces EC death, accelerates ISC aging (as evidenced by ISC hyperproliferation, DNA damage accumulation, and increased centrosome amplification) and affected the adult fly’s survival. In addition, this work provides insight into the essential role of the DDR in the maintenance of niches for stem cell homeostasis under normal conditions, and produced precautionary evidence for the use of inadvertent inhibitors of the DDR such as that observed with some cancer drugs.

## METHODS

### Fly stock

Fly stocks were maintained at 25 °C on standard food under an approximate 12 h/12 h light/dark cycle. Food consisted of 79.2% water, 1% agar, 7% cornmeal, 2% yeast, 10% sucrose, 0.3% bokinin and 0.5% propionic acid. To avoid larval overpopulation in all vials, 50–60 adult flies per vial were transferred to new food vials every 2–3 days for a period of 50–60 days or longer. Transgenic RNAi lines: *UAS-Mre11-RNAi* (#30476, VDRC, Vienna, Austria), *UAS-Rad50-RNAi* (#103394, VDRC), *UAS-Nbs1-RNAi* (#28215, VDRC), *UAS-Nbs1-RNAi* (#28216, VDRC), *UAS-ATM-RNAi* (#22502, VDRC); *UAS-ATM-RNAi* (#108074, VDRC); *UAS-ATR-RNAi* (#11251, VDRC); *UAS-ATR-RNAi* (#103624, VDRC), *UAS-Chk1-RNAi* (#12680, VDRC); *UAS-Chk1-RNAi* (#110076, VDRC), *UAS-Chk2-RNAi* (#110342, VDRC). Temperature-inducible differentiated EC-specific *Myo1A-Gal80^ts^* flies *were obtained from* B.A. Edgar [36]. *Oregon-R flies were used as the wild type. *Myo^ts^>GFP* flies were obtained from a cross of the *Oregon-R* males and Myo1A-GAL4/CyO;UAS-GFP,tub-Gal80^ts^/TM6B (Myo^ts^) females. Myo^ts^>GFP+Mre11i*, *Myo^ts^>GFP+Rad50i*, *Myo^ts^>GFP+Nbs1i*, *Myo^ts^>GFP+ATMi*, *Myo^ts^>GFP+ATRi*, *Myo^ts^>GFP+Chk1i*, *Myo^ts^>GFP+Chk2i* flies were obtained from a cross of the *UAS-Mre11i/UAS-Mre11i, UAS-Rad50i/UAS-Rad50i, UAS-Nbs1i/UAS-Nbs1i, UAS-ATMi/UAS-ATMi, UAS-ATRi/UAS-ATR, UAS-Chk1i/UAS-Chk1i, UAS-Chk2i/UAS-Chk2i* males and *Myo^ts^* females, respectively. The results described in this study were obtained using female flies.

### Temperature-controlled expression

For transgene expression at specific developmental stages, the Gal80^ts^ technique was used [66]. The flies were set up and maintained at 22 °C until adulthood. After maintaining the flies at 29 °C for 4 days, the midguts were dissected and analyzed.

### Immunochemistry

Intact adult guts were dissected and fixed at room temperature. For anti-green fluorescent protein (GFP) antibody staining, the guts were fixed for 1 h in 4% formaldehyde (Sigma-Aldrich, St. Louis, MO, USA). For anti-γH2AvD and Delta antibody staining, the guts were fixed for 30 min in 4% paraformaldehyde (Electron Microscopy Science, USA), dehydrated for 5 min in 50%, 75%, 87.5% and 100% methanol, and rehydrated for 5 min in 50%, 25% and 12.5% methanol in PBST (0.1% Triton X-100 in phosphate-buffered saline) for postfixing. After washing with PBST, the samples were incubated for 1 h with secondary antibodies at 25 °C, washed again in PBST, mounted with Vectashield (Vector Laboratories, Burlingame, CA, USA), and analyzed using a Zeiss Axioskop 2Plus microscope (Carl Zeiss Inc., Göttingen, Germany). PH3^+^ cells were counted in the entire midgut.

### Antisera

The following primary antibodies diluted in PBST were used in these experiments: mouse anti-Delta, mouse anti-Arm (Developmental Studies Hybridoma Bank, Iowa City, IA, USA), 1:200; mouse anti-GFP and rabbit anti-GFP (Molecular Probes, Eugene, OR, USA), 1:1000; rat anti-GFP (Nacalai Tesque Inc., Kyoto. Japan), 1:1000; rabbit anti-γH2AvD (Rockland, Gilbertsville, PA, USA) 1:2000; rabbit anti-pS/TQ (Cell Signaling Technologies, Danvers, MA, USA), 1:1000; rabbit anti-phospho-histone H3 (PH3, Millipore, Billerica, MA, USA), 1:1000; mouse anti-γ-tubulin (Sigma-Aldrich), 1:1000; rabbit anti-β-gal (Upstate Biotechnology Inc., Lake Placid, NY, USA), 1:1000; and anti-CCleaved caspase-3 (Cell Signaling Technologies), 1:1000; rabbit anti-pJNK antibody (Cell Signaling Technologies). The following secondary antibodies diluted in PBST were used: goat anti-rabbit FITC (Jackson ImmunoResearch, West Grove, PA, USA), 1:400; goat anti-rabbit Cy3 (Jackson ImmunoResearch), 1:400; goat anti-mouse FITC (Jackson ImmunoResearch), 1:400; goat anti-mouse Cy3 (Jackson ImmunoResearch), 1:400; goat anti-rat FITC (Jackson ImmunoResearch), 1:400, goat anti-rabbit Alexa Fluor^®^ 647 (Jackson ImmunoResearch), 4′,6-diamidino-2-phenylindole (DAPI, Molecular Probes), 1:1000.

### γ-irradiation

Adult flies were irradiated with a γ-irradiation machine [^137^CS, 21.275tBq (575Ci)] at a dose-rate of 2.55 Gy/min. Following irradiation at 5 Gy dose, irradiated fly and non-irradiated control fly vials were maintained at 25°C, respectively [14].

### Measurement of survival rate

For adult survival analysis, to avoid larval overpopulation in culture vials, 25–30 adult flies were cultured in a vial and transferred to new vials containing fresh food every 2–3 days for a period of 38–40 days or longer. Flies were cultured at 29 °C and surviving flies were counted every two days.

Fly genotypes for survival:

Myo1A-GAL4/+;UAS-GFP,tub-Gal80^ts^/+

Myo1A-GAL4/+;UAS-GFP,tub-Gal80^ts^/ UAS-Mre11-RNAi

Myo1A-GAL4/ UAS-Rad50-RNAi;UAS-GFP,tub-Gal80^ts^/+

Myo1A-GAL4/+;UAS-GFP,tub-Gal80^ts^/UAS-Nbs1-RNAi

Myo1A-GAL4/+;UAS-GFP,tub-Gal80^ts^/ UAS-ATM-RNAi

Myo1A-GAL4/UAS-ATR-RNAi;UAS-GFP,tub-Gal80^ts^/+

Myo1A-GAL4/+;UAS-GFP,tub-Gal80^ts^/ UAS-Chk1-RNAi

Myo1A-GAL4/UAS-Chk2-RNAi;UAS-GFP,tub-Gal80^ts^/+

### Paraquat feeding assay

Three-day-old flies were cultured in standard media for 6 h at 29 °C. And then, flies were fed 2 mM paraquat (PQ, methyl viologen, Sigma-Aldrich) in 5% sucrose media for 18 h at 29 °C. The midgut of the flies were analyzed by immunostaining.

### Quantitative analysis

To quantitatively analyze PH3-positive cells, the number of PH3-positive cells in the whole gut was counted. To quantitatively analyze centrosome amplification, the number of γ-tubulin stained spots per PH3-positive cell in the whole midguts was determined. Quantified data are expressed as the mean±SE. Significant differences were identified using the Student’s *t*-test. Sigma Plot 10.0 (Systat Software Inc., San Jose, CA, USA) was used for analysis of standard error.

## Supplementary Material

Supplementary File
