# Supplementary material for: Deficiency in DNA damage response of enterocytes accelerates intestinal stem cell aging in Drosophila
Source: Aging (Albany NY). 2018 Mar 7;10(3):322–38. doi: 10.18632/aging.101390 (PMC5892683; doi:10.18632/aging.101390)
Supplement: Supplementary File [file aging-10-101390-s001.pdf]

SUPPLEMENTARY MATERIAL

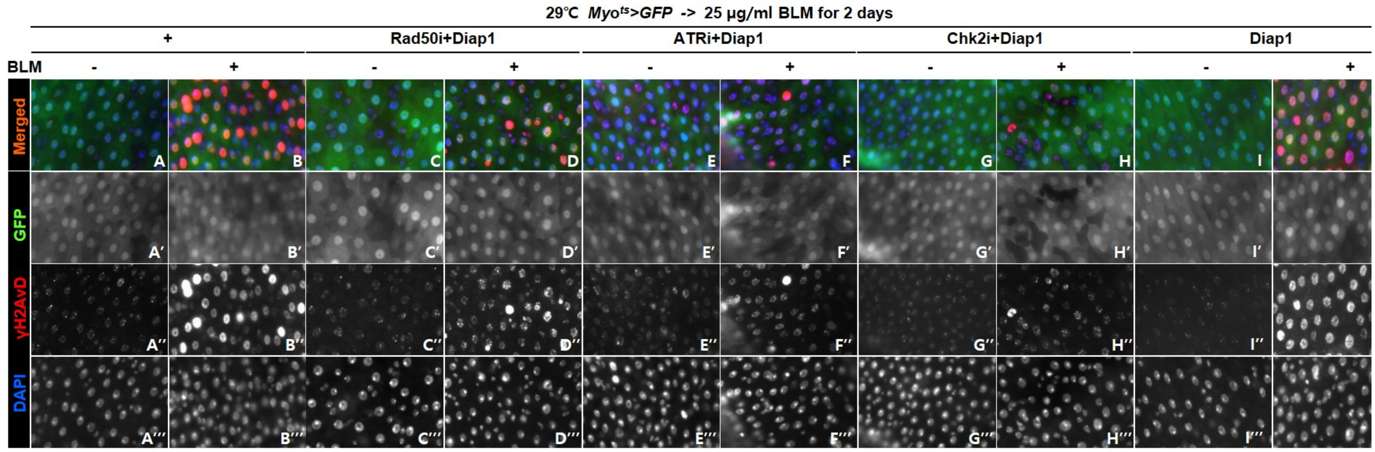

Supplementary Figure 1. Confirmation of combination with DDR-related factors knockdown and Diap1 overexpression. Flies carrying *Myo*^{ts}*>GFP*, *Myo*^{ts}*>GFP+Rad50i*, *Myo*^{ts}*>GFP+Rad50i+Diap1*, *Myo*^{ts}*>GFP+ATRi*, *Myo*^{ts}*>GFP+ATRi+Diap1*, *Myo*^{ts}*>GFP+Chk2i*, *Myo*^{ts}*>GFP+Chk2i+Diap1*, or *Myo*^{ts}*>GFP+Diap1* genotypes were fed media with 25 µg/ml Bleomycin at 29 °C for 2 days. The guts of flies were dissected and labeled with anti-GFP (green) and anti-γH2AvD (red) antibodies and DAPI (blue). A'-A''', B'-B''', C'-C''', D'-D''', E'-E''', F'-F''', G'-G''', H'-H''',I'-I''', and J'-J''' were gray scale image of in A-J, respectively. Original magnification is 400×.

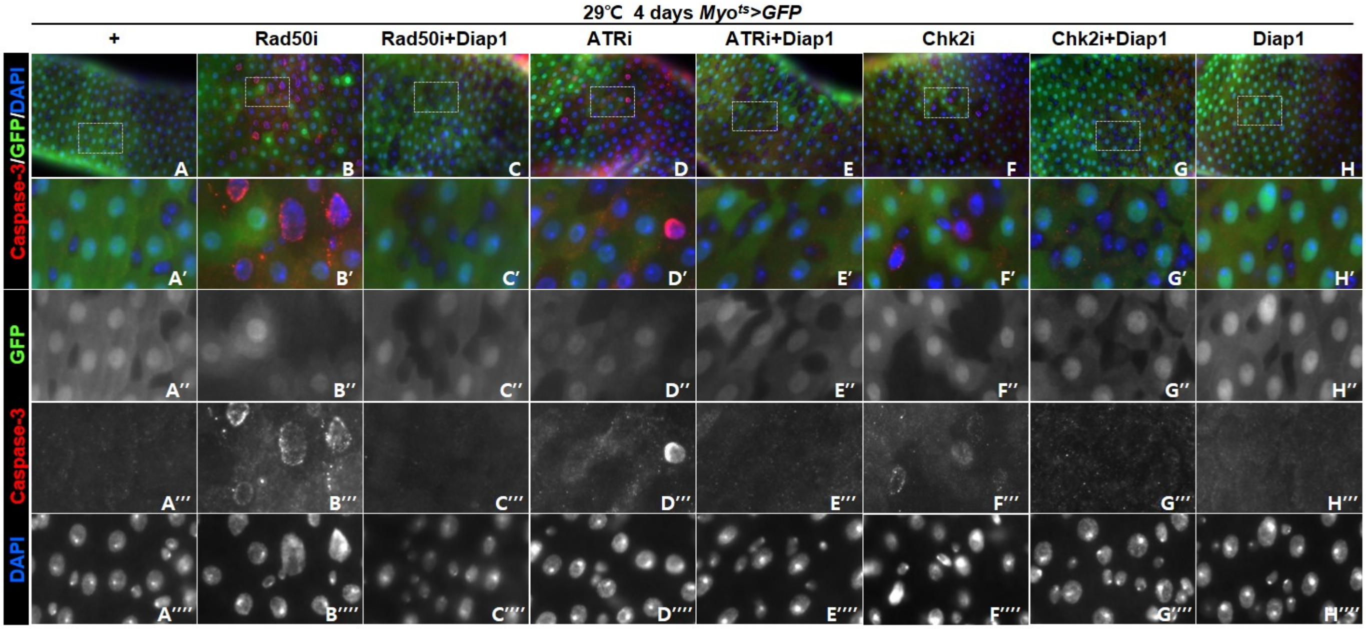

Supplementary Figure 2. Effect of Diap1 overexpression on EC-specific knockdown of DDR-related factors-induced of EC death. Flies carrying *Myo*^{ts}*>GFP*, *Myo*^{ts}*>GFP+Rad50i*, *Myo*^{ts}*>GFP+Rad50i+Diap1*, *Myo*^{ts}*>GFP+ATRi*, *Myo*^{ts}*>GFP+ATRi+Diap1*, *Myo*^{ts}*>GFP+Chk2i*, *Myo*^{ts}*>GFP+Chk2i+Diap1*, or *Myo*^{ts}*>GFP+Diap1* genotypes were cultured at 29 °C for 4 days. The guts of flies were dissected and labeled with anti-GFP (green) and anti-Cleaved Caspase-3 (red) antibodies and DAPI (blue). A'-A''', B'-B''', C'-C''', D'-D''', E'-E''', F'-F''', G'-G''', and H'-H''' were enlarged image of white square in A-H, respectively. A''-A'''', B''-B'''', C''-C'''', D''-D'''', E''-E'''', F''-F'''', G''-G'''', and H''-H'''' were gray scale image of in A'-H', respectively. Original magnification is 400×.

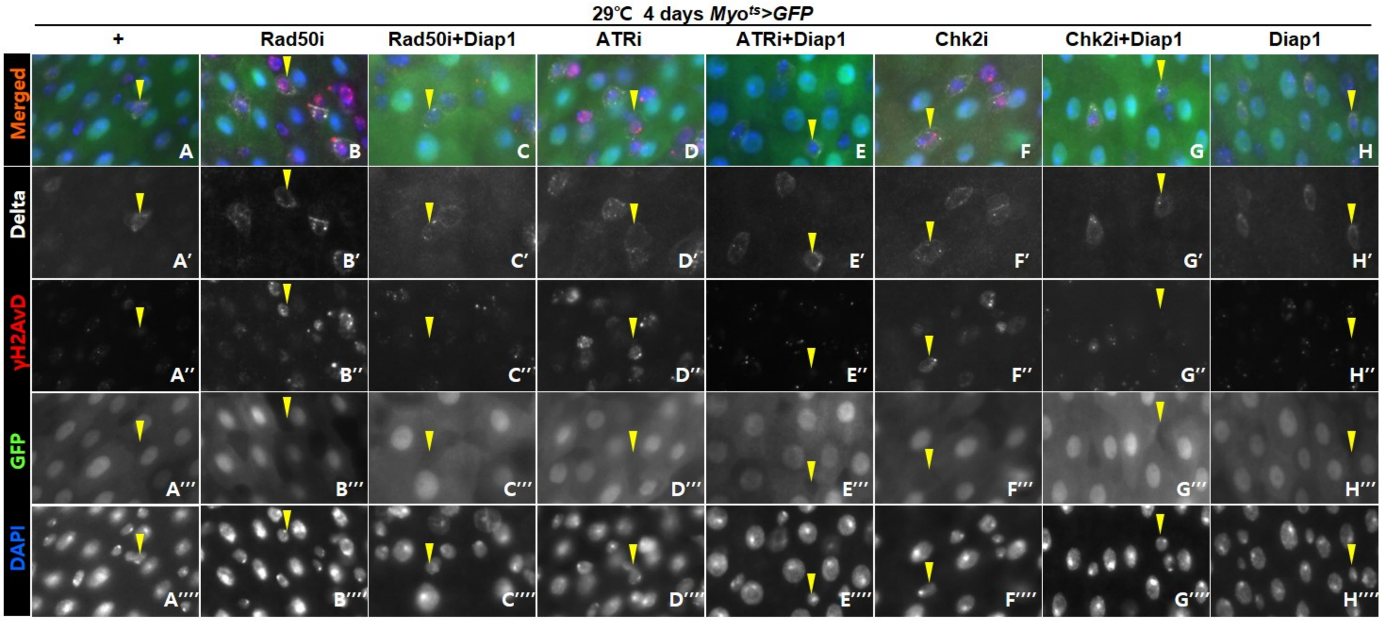

Supplementary Figure 3. Effect of Diap1 overexpression on EC-specific knockdown of DDR-related factors-induced DNA damage accumulation in ISCs. Flies carrying *Myo^{ts}>GFP*, *Myo^{ts}>GFP+Rad50i*, *Myo^{ts}>GFP+Rad50i+Diap1*, *Myo^{ts}>GFP+ATRi*, *Myo^{ts}>GFP+ATRi+Diap1*, *Myo^{ts}>GFP+Chk2i*, *Myo^{ts}>GFP+Chk2i+Diap1*, or *Myo^{ts}>GFP+Diap1* genotypes were cultured at 29 °C for 4 days. The guts of flies were dissected and labeled with anti-GFP (green), anti-Delta (white), and anti-γH2AvD (red) antibodies and DAPI (blue). A'-A'''', B'-B'''', C'-C'''', D'-D'''', E'-E'''', F'-F'''', G'-G'''', and H'-H''' were gray scale image of in A-H, respectively. Yellow arrow heads indicate Delta$^+$ cell. Original magnification is 400×.

SUPPLEMENTARY MATERIALS AND METHODS

Flies

UAS-Diap1 were kindly provided by the Bloomington Stock Center.

Fly genotypes

Myo1A-GAL4/+;UAS-GFP,tub-Gal80^{ts}/+
Myo1A-GAL4/UAS-Rad50-RNAi;UAS-GFP,tub-Gal80^{ts}/+
Myo1A-GAL4/UAS-Rad50-RNAi;UAS-GFP,tub-Gal80^{ts}/UAS-Diap1
Myo1A-GAL4/UAS-ATR-RNAi;UAS-GFP,tub-Gal80^{ts}/+
Myo1A-GAL4/UAS-ATR-RNAi;UAS-GFP,tub-Gal80^{ts}/UAS-Diap1
Myo1A-GAL4/UAS-Chk2-RNAi;UAS-GFP,tub-Gal80^{ts}/+
Myo1A-GAL4/UAS-Chk2-RNAi;UAS-GFP,tub-Gal80^{ts}/UAS-Diap1
Myo1A-GAL4/+;UAS-GFP,tub-Gal80^{ts}/UAS-Diap1

Bleomycin feeding assay

Three-day-old flies were fed 25 ㎍/㎖ Bleomycin (Sigma-Aldrich) in normal media for 2 days at 29 °C.
